# 3D Femtosecond Laser Beam Deflection for High‐Precision Fabrication and Modulation of Individual Voxelated PCM Meta‐Atoms

**DOI:** 10.1002/advs.202413316

**Published:** 2025-01-13

**Authors:** Weina Han, Donghui Wei, Biye Peng, Jianhui Jiang, Jintao Tong, Zhehao Xu, Xueyan Zou, Jie Hu, Qian Cheng, Lan Jiang

**Affiliations:** ^1^ Laser Micro/Nano Fabrication Laboratory School of Mechanical Engineering Beijing Institute of Technology Beijing 100081 P. R. China; ^2^ Beijing Institute of Technology Chongqing Innovation Center Chongqing 401120 P. R. China

**Keywords:** 3D beam deflection, fabrication, femtosecond laser, modulation, PCM meta‐atoms

## Abstract

Optical metasurfaces have found widespread applications in the field of optoelectronic devices. However, achieving dynamic and flexible control over metasurface functionalities, while also developing simplified fabrication methods for metasurfaces, continues to pose a significant challenge. Here, the study introduces a PCM‐only metasurface that exclusively consists of voxel units crafted from different phases of phase‐change materials. Micro‐nano regions, with varying phase states, are directly utilized as resonant elements and embedded in the material, forming the metasurface voxel meta‐atoms. By manipulating the morphology, size, and arrangement of these meta‐atoms, the study achieves both the fabrication and modulation of the PCM‐only metasurface. Additionally, a 3D high‐precision voxelated beam deflection method based on femtosecond laser phase modulation is introduced to streamline the fabrication and modulation of PCM‐only metasurface. By using a spatial light modulator to load the blazed grating phase manipulation beam for precise deflection, high‐precision deflection processing of individual meta‐atom on metasurfaces can be achieved, with a deflection accuracy of up to 200nm. By loading Fresnel phase manipulation beams to move along the z‐axis, perfect modulation of PCM sub‐meta‐atoms can be achieved. The 3D femtosecond laser beam deflection technology will bring many potential application opportunities in the fields of optoelectronic device fabrication and functional control.

## Graphical abstract

The study presents a PCM‐only metasurface that is constructed entirely of voxel units comprising various stages of phase‐change materials, and introduces a 3D, high‐precision beam deflection method that utilizes femtosecond laser phase modulation and achieves a processing accuracy of 200nm. This deflection technique enables precise manipulation of individual PCM meta‐atoms, thus facilitating the high‐precision fabrication and modulation of metasurfaces.

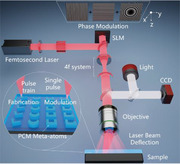

## 1 Introduction

As an array of artificial structural units at a subwavelength scale, metasurface can flexibly manipulate electromagnetic waves and have important application value in optoelectronic devices such as optical imaging^[^ ^1^ ^]^ and information storage.^[^ ^2^ ^]^ Traditional metasurface processing and manufacturing methods mostly use electron beam lithography,^[^ ^3^ ^]^ ion beam etching,^[^ ^4^ ^]^ nanoimprinting, and self‐assembly.^[^ ^5^, ^6^ ^]^ However, these manufacturing methods have problems such as high cost, complex process, insufficient flexibility, and limited material selection, which have seriously restricted the research and application value of metasurface photonic devices.^[^ ^7^, ^8^, ^9^ ^]^ Many studies are dedicated to the flexible fabrication and modulation of high‐precision meta‐atoms. For example, high precision laser printing can be achieved by adjusting the laser pulse energy to reshape the metasurface structure,^[^ ^10^, ^11^ ^]^ while information storage is enabled by altering nanoparticle properties and shape through changes in laser energy and polarization.^[^ ^12^ ^]^ In addition, once traditional metasurface photonic devices are manufactured, their unit structure and properties cannot be changed and their functions are fixed. However, more and more scenarios require that the functions of devices can be dynamically controlled.^[^ ^13^ ^]^ New generation photonic devices such as spatial light modulators,^[^ ^14^ ^]^ tunable lenses,^[^ ^15^ ^]^ and dynamic holograms^[^ ^16^ ^]^ need to meet programmable functional requirements, which require metasurface to be able to flexibly and accurately dynamically control each independent meta‐atom.

In order to realize the dynamic control function of metasurfaces, active materials are usually introduced during the design of metasurfaces, and the dynamic control of device functions can be achieved by controlling the properties of active materials.^[^ ^17^ ^]^ Phase‐change material (PCM) is functional material capable of reversible phase transitions under laser, heat, or electrical stimulation. There are drastic differences in the optical and electrical properties of PCM such as refractive index and conductivity in different phases. This unique property makes PCM have important application value in the field of dynamically reconfigurable metasurface.^[^ ^12^, ^18^, ^19^, ^20^, ^21^ ^]^ At present, PCM mainly exists in the form of active tuning layers or composite superposition with other materials in metasurfaces, and dynamic control of function is achieved through heating or electric pulse stimulation.^[^ ^22^, ^23^, ^24^, ^25^ ^]^ However, the complexity of metasurface structures composed of multiple materials poses challenges in fabrication, and thermal and electrical control limits the flexibility and response speed of device dynamic control. Therefore, to facilitate the flexible and widespread application of PCM in metasurface photon devices, it is crucial to develop simpler fabrication and more flexible modulation methods for PCM.

Due to its ultra‐short pulse duration and ultra‐high peak intensity, ultrafast laser can accurately and quickly perform localized crystallization or amorphization of PCM, and has unique advantages in the fabrication and modulation of PCM.^[^ ^26^, ^27^, ^28^ ^]^ However, the feature size of micro‐nano resonant units in metasurfaces are typically in the submicron or even nanometer scale. Traditional laser direct writing techniques are limited by the precision of mechanical components such as translation stages and mechanical shutters, making it difficult to guarantee fabrication accuracy. Compared with the laser direct writing processing method, the beam deflection fabrication method using the scanning system can improve the movement speed and accuracy of the focused spot. However, since the scanning system usually requires the use of a special field mirror to focus the beam, its focusing ability is lower than for high numerical aperture objectives, the focal spot size is usually larger than 10 microns, which cannot meet the requirements of submicron or nanometer‐level fabrication accuracy.^[^ ^29^ ^]^ By spatially shaping the beam, microstructures of various complex shapes can be directly manufactured under single or multiple pulse exposures, significantly improving the efficiency and resolution of metasurface fabrication. However, the energy fluctuations caused by the noise between the shaped light fields There are still restrictions on the processing and modulation application of shaped optical fields in PCM.^[^ ^30^, ^31^, ^32^, ^33^ ^]^ Therefore, in order to apply ultrafast laser in the field of metasurface fabrication, how to achieve fine manipulation of ultrafast laser with small spots has become the key to the problem.

In this work, we propose a PCM‐only metasurface constructed by patterning crystallized/amorphized voxel meta‐atoms, and a high‐precision voxelated beam 3D deflection fabrication technology based on femtosecond laser phase modulation to achieve high‐precision, high‐consistency, and high‐flexible fabrication and modulation of the metasurface. We directly use micro‐nano regions of different phases as resonant units embedded inside the material to form metasurface voxel meta‐atoms, and by modulating the morphology, size, arrangement, and phase of these units, we achieve the synergistic fabricating and dynamic control of PCM‐only metasurface. The result is a significant simplification of the metasurface fabrication and modulation process. We use high numerical aperture objectives combined with laser phase modulation to achieve precise manipulation of laser spots. By loading the blazed grating phase and Fresnel lens phase through the spatial light modulator to achieve precise deflection of the light beam in the propagation direction. And we use the 4f system to transport the phase to ensure that the modulation plane coincides with the objective lens entrance pupil plane, so that the deflection of the light beam through the objective lens can achieve the shortest optical path, thereby ensuring the accuracy of the beam deflection. Then, by pixelating arbitrary patterned micro‐nano structures, calculating he blazed grating deflection phase, and generating deflection paths, we ultimately achieve high‐precision deflection fabrication and modulation of surface micro‐nano structures. In addition, we achieve up to 20×20 parallel processing by superimposing multiple light spot phases, which greatly improves the fabrication and modulation efficiency.

## 2 Results and Discussion

### 2.1 All‐Optical Fabrication‐Modulation of Reconfigurable PCM‐Only Metasurface

**Figure 1:**
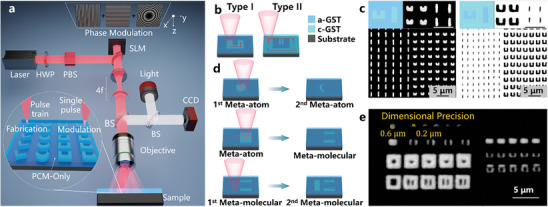
High‐precision voxelated beam deflection technology based on femtosecond laser phase modulation is used for the fabrication and modulation of PCM‐only metasurface. a) Schematic diagram of high‐precision voxelated beam deflection fabrication‐modulation method based on femtosecond laser phase modulation. b) Schematic diagram of two types of fabrication methods for PCM‐only Metasurface. c) Optical micrographs of the processing patterns of the two types of processing methods in inset (b), the left picture corresponds to “Type I”, and the right picture corresponds to “Type II”. The upper illustration shows a schematic diagram of the processed pattern on the left and a magnified optical micrograph on the right. Scale bar is 5 µm. d) PCM‐Only metasurface modulation schematic. e) Optical micrograph of PCM‐only metasurface modulation.

**Figure** **1**a shows a high‐precision voxelated beam deflection fabrication‐modulation method based on femtosecond laser phase modulation. Laser phase control can achieve precise 3D control of the laser focus, in which the blazed grating phase can realize the deflection of the beam in the propagation direction, the Fresnel lens phase realizes the movement of the focused spot on the z‐axis, and the laser phase is precisely controlled by a reflective phase‐type spatial light modulator (SLM). In order to ensure the accuracy of beam deflection, the laser modulation plane needs to coincide with the entrance pupil plane of the processing objective. The 4f system can realize phase transfer to ensure that the modulation plane coincides with the entrance pupil plane of the objective lens. Therefore, the deflection of the beam through the objective lens can achieve the shortest optical path, thereby ensuring the accuracy of the beam deflection. When a blazed grating is loaded on the SLM to achieve beam deflection, the degree of beam deflection is determined by parameters such as the pixel resolution, number of pixels, and wavelength of the SLM. The maximum deflection angle θ_*max*_ is as follows:

(1) $$\theta_{max}=arcsin\left(\frac{\lambda }{2d_{pix}}\right)$$

where λ is the wavelength of incident laser and *d_pix_* is the pixel pitch of SLM. The minimum deflection angle θ_*min*_ is as follows:

(2) $$\theta_{min}=arcsin\left(\frac{\lambda }{d_{Length}}\right)$$

where *d_Length_* is the effective working side length of the SLM. Therefore, the deflection spacing can be determined by the deflection angle and the focal length of the objective lens:

(3) $$\Delta_{d}=tan\;\;\left(\theta \right)\cdot f$$

According to our experimental conditions (λ = 1.03 µm, *f* = 3.6 mm, *d_pix_* = 8 µm, *d_Length_* = 8 × 1080 = 8640 µm), he minimum deflection distance is 0.4 µm. In addition, the z‐axis movement distance d of the laser focus is as follows:

(4) $$\Delta d=\left|\frac{f_{fresnel}\times f_{objective}}{f_{fresnel}+f_{objective}}-f_{objective}\right|$$

where *f_fresnel_* is the focal length of the loaded Fresnel phase, and *f_objective_* is the focal length of the objective lens. In addition, we simulated and verified the phase‐controlled focused light field, as shown in Figure S1 (Supporting Information), to verify the feasibility of this fabrication technology.

As shown in Figure 1b, we have developed two different PCM‐Only Metasurface fabrication methods based on the beam deflection technology of femtosecond laser phase modulation: filling the structure composed of voxel units (Type I) and filling periodic background (Type II). Type I method involves femtosecond laser directly acting on the designed patterned structure, while Type II method is that laser acts on the periodic background and retains part of the patterned structure. Figure 1c shows the rod‐shaped structure and split ring structure fabricated by the two types. For these two types, we can perform high‐flexibility processing of single “meta‐atom” configuration and “meta‐molecule” configuration composed of multiple “meta‐atom” respectively. And in Figure 1d, by adjusting the fabrication parameters of the laser, we can perform localized writing and erasing of the processed micro‐nanostructure units, perform high‐precision modulation at the “sub‐meta‐atomic” level, and realize the flexible conversion between “meta‐atom” and “meta‐molecule”. Figure 1e shows the modulation of the circular and rectangular structures fabricated by the two types of methods. Through localized erasure, the circular structure is modulated into a crescent‐shaped structure, and the distance‐shaped structure is transformed into a dual‐antenna structure. We further wrote on the basis of the dual‐antenna structure, this structure can also be modulated into a three‐antenna structure. And because we used a NA 0.8 high numerical aperture objective lens, we achieved the feature size of a 600 nm small spot and the refined fabrication of a 200 nm deflection spacing, which provides strong technical support for the application of femtosecond lasers in sub‐wavelength unit fabrication.

### 2.2 PCM‐Only Metasurface Fabrication Based on the Beam Deflection Technology of Femtosecond Laser Phase Modulation

**Figure 2:**
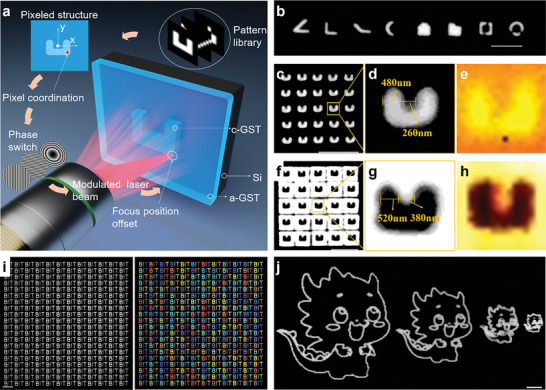
Schematic diagram and optical micrograph of high‐precision voxelated beam deflection based on femtosecond laser phase modulation. a) Schematic diagram of high‐precision voxelated beam deflection based on femtosecond laser phase modulation. b) Optical micrographs of various metasurface structural units fabricated by femtosecond laser based on phase modulation. c–e) Pattern fabricated by “Type I” method, and c‐d are optical micrographs, e is a Raman image. f–h) Pattern fabricated by “Type II” method, and f‐g are optical micrographs, h is a Raman image. j) Confocal pattern of crystal structure of cartoon dragon image. Scale bar is 5 µm.

In **Figure** **2**a, we build a pattern library for the metasurface structure unit. Due to the fact that the metasurface unit structure is usually a 3D pattern, we convert it into pixel coordinates through pixelation processing, and generate a pixel coordinate path through path planning. Then, the deflection phase of each pixel point on the path is converted and loaded onto the spatial light modulator (SLM). When the laser passes through the SLM, the deflection phase of the beam is loaded, and then the phase is transported through the 4f lens system, so that the deflection phase acts on the entrance pupil plane of the objective lens. Under the action of the objective lens, the focus on the focal plane is offset to achieve single pixel processing of the patterned structure. Through the above steps, the operation of all pixel points on the path can be completed, and finally the deflection processing of any patterned structure can be achieved. Based on this, we processed a variety of complex configurations of PCM‐only metasurface unit structures (Figure 2b), proving that this technology can ensure the accuracy of feature size while having extremely high flexibility and can be applied to the processing of any patterned structure. Figure 2e,h are Raman mapping images, where different colors represent different properties of the material. As shown in Figure S2 (Supporting Information), we extracted Raman spectra of the fabricated and non‐fabricated areas, demonstrating that we have achieved high‐precision phase transformation fabrication.

The processing accuracy is determined by the spot size and the minimum deflection spacing of the beam. In order to achieve high‐precision and high‐quality super‐surface structure processing, we studied the parameters of the single‐point crystallization modified circle of a 50 nm GST film on a single‐crystal silicon substrate under an objective lens with an NA value of 0.8 and a magnification of 50× (Figure S2, Supporting Information). After controlling the laser single pulse energy, deflection spacing, and other parameters, we fabricated the groove‐type pattern array with a period of 3 µm using two types of methods (Figure 2c–h). Among them, the crystallization line width of the two arms of the type I groove can be less than 500 nm, and the distance between the two arms is less than 300 nm. The crystallization size between the two arms of the type II groove can be less than 400 nm, and the amorphous line width of the two arms is ≈500 nm, which has extremely high feature size accuracy.

To assess the fabrication quality of this technology in large‐area applications, we calculated the overlap rate of individual fabrication units as a metric for fabrication consistency. (Refer to Figure S4, Supporting Information for details on the detection principle of fabrication consistency). In Figure 2i, we fabricated a large‐area array of complex “BIT” pattern units, achieving an overlap rate exceeding 95%. Furthermore, this technology ensures exceptional precision in small areas and facilitates large‐area pixel fabricating with maintained detail accuracy over a substantial range. In Figure 2j, we applied this technology to fabricate the crystallized structures of cartoon dragon images varying in size. The line widths of these structures are less than 600 nm, with the largest dimension measuring 36 µm and the smallest ≈6 µm. This technology demonstrates a remarkable tolerance and flexibility within the fabricating range, underscoring its adaptability to diverse applications. Notably, even in extensive fabricating areas, this technology ensures fabrication accuracy, thereby catering to various application requirements. Despite the aforementioned advantages of high precision, consistency, and flexibility, the fabrication rate of this technology is constrained by hardware factors, including the refresh rate of the spatial light modulator and crystal phase loading time. To enhance fabricating efficiency, we introduced a novel method leveraging femtosecond laser spatial shaping and multi‐spot parallel scanning. As shown in Figure S5 and Movie S1 (Supporting Information), transforming a single femtosecond laser beam into a multi‐spot laser field significantly boosts fabricating efficiency in a geometric manner. Through multi‐spot phase loading, we achieved up to 20 × 20 parallel fabrication, resulting in a remarkable 400‐fold efficiency enhancement.

### 2.3 All‐Optical Modulation of Reconfigurable PCM‐Only Metasurface

**Figure 3:**
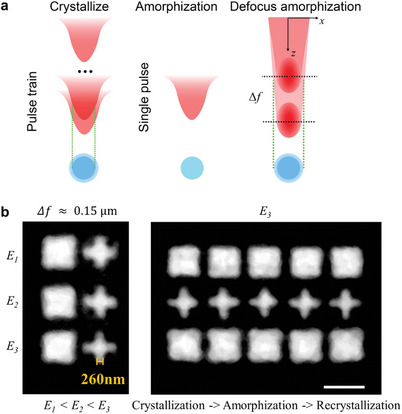
Defocused reversible phase change based on phase modulation. a) Schematic diagram of defocused reversible phase change based on phase modulation. b) Optical micrographs of the parameters of the GST defocus reversible modulation study. Scale bar is 2 µm.

Femtosecond lasers enable swift, precise, and adaptable reversible modulation of GST, facilitating dynamic adjustability in PCM‐only metasurfaces. However, the processes of GST crystallization and amorphization exhibit distinct differences. The unequal areas of crystallized and amorphized regions post‐femtosecond laser pulses hinder perfect full‐coverage reversible phase conversion, posing a challenge for precise reversible metasurface modulation. Hence, comprehending the GST phase conversion mechanism and conducting thorough parameter studies on its crystallization and amorphization are crucial for achieving dynamic reversible modulation in PCM‐only metasurfaces. As depicted in **Figure** **3**a, femtosecond laser‐induced GST crystallization necessitates multi‐pulse induction, whereas amorphization is achieved with a single pulse, highlighting distinct thresholds for both processes. The amorphization threshold (*E_a_*) surpasses the crystallization threshold (*E_c_*). Given the varying energy and pulse requirements for GST crystallization and amorphization, their respective areas under femtosecond laser induction differ as well. To align the crystallization and amorphization areas, we introduce Fresnel phase through femtosecond laser phase modulation for precise defocusing control of laser focus on the z‐axis, enabling precise defocus control for achieving flawless reversible modulation. Figure 3b illustrates the processing of a unit area within a crystallized small rectangular metasurface. By introducing a slight defocus, we manipulate the amorphization area through variations in single pulse energy, facilitating specific parameter investigations. In the left panel of Figure 3c, upon introducing a defocus of Δ*f*= 0.15 µm, the area of amorphous erasure gradually expands with an increase in single pulse energy (*E_1_* = 1.8*E*, *E_2_* = 2*E*, and *E_3_* = 2.2*E*, where *E* is the energy of a single crystalline pulse, with *E* = 1.55 nJ). When the energy increases to *E_3_*, we successfully achieve a clean amorphous erasure of the four corners of the small rectangle, transforming it into a small cross. As demonstrated in the right Figure 3b, multiple cycles of crystallization, amorphization, and recrystallization were conducted, confirming the ability to achieve stable modulation of the PC‐only metasurface through the introduction of a small defocus adjustment facilitated by the Fresnel phase.

**Figure 4:**
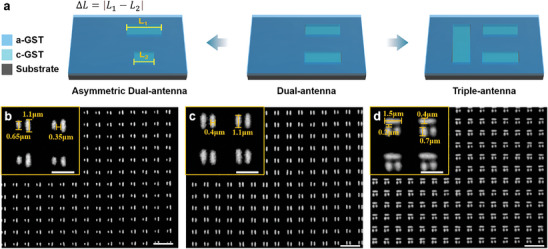
Dual‐antenna PCM‐only metasurface dynamic modulation. a) Schematic diagram of dual‐antenna PCM‐only metasurface modulation. The structural period is 2 µm, the GST film thickness is 50 nm, and the substrate is fused quartz. b–d) Optical micrographs of dual‐antenna PCM‐only metasurface fabrication and modulation. Scale bar is 5 µm.

Metasurfaces are planar arrays made of subwavelength‐sized meta‐atoms, capable of flexibly manipulating parameters like amplitude, phase, and polarization of optical fields. They have significant applications in optical imaging, communication, and displays.^[^ ^34^, ^35^, ^36^ ^]^ As shown in **Figure** **4**a, we devised a PCM‐based dual‐antenna metasurface that can be dynamically tuned by localized erasure to an asymmetric configuration or by localized writing to a triple‐antenna setup, enabling dynamic optical response adjustment. As illustrated in Figure 4b–d, we fabricated a large‐area PCM‐only metasurface array featuring a dual‐antenna structure, leveraging high‐precision femtosecond laser phase modulation for voxelated beam deflection. This enabled precise modulation to individual meta‐atoms, facilitating flexible transitions between distinct “meta‐atoms” and “meta‐molecules”.

**Figure 5:**
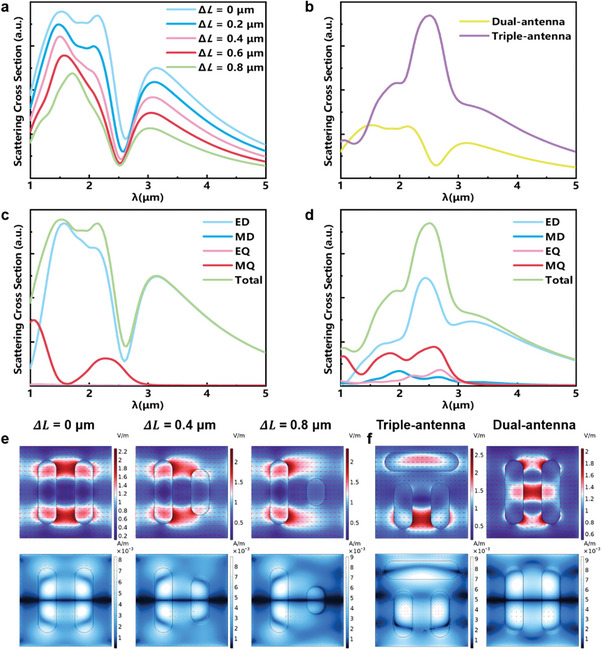
Simulation of the dynamic modulation of the scattering spectrum of the dual‐antenna PCM‐only metasurface. a) Modulated scattering spectrum of the dual‐antenna metasurface. b) Modulated scattering spectrum of the dual‐antenna and triple‐antenna metasurfaces. c) Multipole decomposition of the dual‐antenna metasurface. d) Multipole decomposition of the triple‐antenna metasurface. e) Cross‐sectional electromagnetic field of the dual‐antenna metasurface, the length difference between the two antennas is Δ*L* = 0 µm, Δ*L* = 0.4 µm, Δ*L* = 0.8 µm, λ = 2.12 µm respectively. f) Cross‐sectional electromagnetic field of the dual‐antenna and triple‐antenna metasurfaces, λ = 2.52 µm.

We used COMSOL Multiphysics to simulate the scattering spectrum and modulation process of a dual‐antenna PCM‐only metasurface, as shown in **Figure** **5**. Figure 5a illustrates that dynamic modulation of the scattering spectrum is achievable by adjusting the length difference (Δ*L*) between the two antennas. At Δ*L* = 0 µm, the dual antenna exhibits distinct scattering peaks at 1.5, 2.3, and 3.2 µm. As Δ*L* increases, the symmetry of the dual antenna is disrupted, causing the scattering peak at 2.3 µm to rapidly diminish and eventually disappear. Additionally, the peaks at 1.5 and 3.2 µm gradually weaken, with their positions undergoing red‐shift and blue‐shift, respectively. Figure 5b demonstrates that incorporating an additional antenna onto the dual‐antenna configuration results in a distinct spectral response. We simulated and compared the scattering spectra of dual‐antenna and triple‐antenna metasurfaces. At 2.52 µm, the dual‐antenna configuration exhibits low scattering intensity, whereas the triple‐antenna configuration displays a pronounced scattering peak. Furthermore, we conducted multipole decomposition and cross‐sectional electromagnetic field simulations to elucidate the resonant modes of both the dual‐antenna and triple‐antenna structures based on their scattering spectra.^[^ ^37^ ^]^ As depicted in Figure 5c,d, the dual‐antenna structure exhibits a notable scattering peak enhancement at 1.52 µm due to the electric dipole (ED) contribution, which diminishes with increasing wavelength. Subsequently, the magnetic quadrupole (MQ) contribution intensifies, leading to a re‐emergence of the scattering enhancement peak at 2.12 µm. As wavelength continues to increase, the contribution of both ED and MQ gradually diminishes, leading to scattering suppression at 2.52 µm. The triple‐antenna structure exhibits a strong resonance peak at 2.52 µm, stemming from the synergistic action of the ED and MQ modes. Figure 5e illustrates that as Δ*L* augments in the dual‐antenna structure, both ED and MQ modes progressively weaken at 2.12 µm, elucidating the diminished scattering spectrum observed. Figure 5f ’s electromagnetic field cross‐sectional diagram further validates this, revealing a dominant ED mode at 2.52 µm for the triple‐antenna structure, in contrast to the MQ dominance in the dual‐antenna structure, aligning with the multipole decomposition results from Figure 5c,d.

### 2.4 Dynamically Reconfigurable Photonic Devices

**Figure 6:**
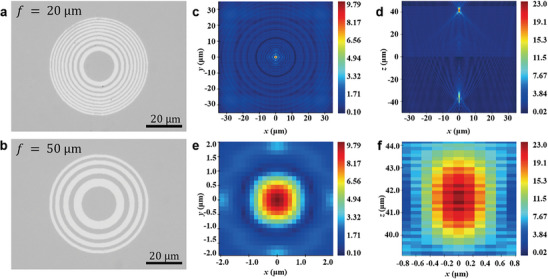
Reconfigurable Fresnel zone plate. a) Schematic diagram of Fresnel zone plate and design method. b) Optical micrograph of a Fresnel zone plate based on GST. c–f) Optical field simulation of Fresnel zone plate with a focal length of 20 microns. Among them, insets (c,d) are the interface diagrams of the light fields in the xy‐plane and xz‐plane. Insets (e,f) are the cross‐sectional diagrams of the light fields in the xy‐plane and xz‐plane at the focal point.

Metasurface photonic elements featuring adjustable parameters, such as variable focal length lenses and dynamic holographic devices, have the capability to alter their functionalities upon light stimulation, enabling diverse practical applications.^[^ ^38^, ^39^, ^40^, ^41^ ^]^ Leveraging high‐precision pixelated beam deflection technology through femtosecond laser phase modulation, we fabricate the GST by converting the metasurface structure into pixelated coordinates and designing the deflection trajectory of the femtosecond laser pulse. These elements can be inscribed, erased, and re‐inscribed into 2D second‐order phase change patterns, facilitating all‐optical fabrication and modulation of tunable metasurface photonic devices. **Figure** **6**a,b demonstrate the fabrication of Fresnel zone plates operating at 1030 nm with focal lengths of 20 µm and 50 µm. This was achieved by calculating the two‐bit phase profile of the meta lens, converting it to pixel coordinates, and subsequently applying pixel‐level deflection‐patterned crystallization to the GST material. Additionally, dynamic reconfiguration of the focal length of the Fresnel zone plate device is achievable via write‐erase‐write cycles (see Figure S6 and Movie S2, Supporting Information). Figure 6c–f presents the simulated light field propagation of the PCM‐only Fresnel zone plate using FDTD. The significant difference in refractive index between the crystalline and amorphous phases of GST results in the incident plane wave being shaped into a Fresnel wavefront by the zone plate upon transmission, ultimately producing a focusing effect.

**Figure 7:**
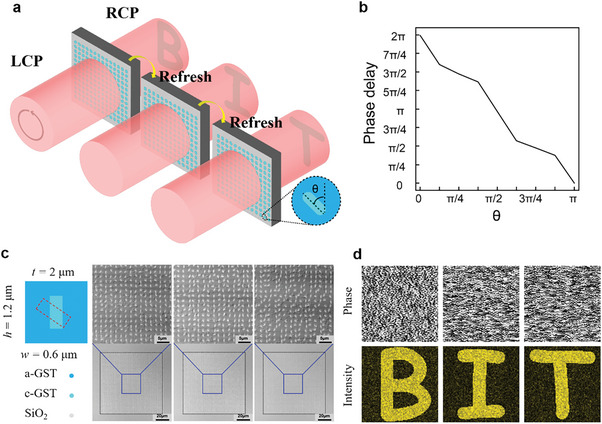
Dynamically reconfigurable holographic metasurface. a) Schematic diagram of dynamically reconfigurable holographic metasurface. b) Subwavelength antenna heading angle and phase delay. c) Holographic phase image and simulated light field intensity image. d) Laser confocal images of dynamically reconfigurable holographic PCM‐only metasurface fabrication.

In **Figure** **7**a, we present a schematic of a dynamically reconfigurable holographic metasurface that utilizes a PCM‐only metasurface, with the holographic phase encoded into the GST. Upon incidence of left‐handed (or right‐handed) circularly polarized light through the metasurface, the polarization conversion of the metasurface units results in a corresponding phase delay, projecting the designed light field pattern after a specified propagation distance. Dynamic regulation of the metasurface holographic device is achieved through erasing and rewriting the encoded hologram to create a new one. We designed and calculated three holographic phase images, each corresponding to the letters “B”, “I”, and “T” in their light field propagation. The dynamic reconfigurable projection of the holographic metasurface device for the three letters is achieved through the iterative process of write‐erase‐write. The phase delay of the subwavelength “volume metasurface” unit was simulated using FDTD. Figure 7b demonstrates a high linear correlation between the antenna orientation angle and the phase delay, enabling a 0–2π phase delay across an angle range of 0‐π. The left figure in Figure 7c shows a metasurface hologram processed using femtosecond laser phase regulation, consisting of small rectangles with dimensions of 0.6 µm width and 1.2 µm length, based on beam deflection technology. The hologram is refreshed by comparing the refreshed version with the current one, erasing and rewriting the differing parts. The holograms necessary for projecting the three letters were calculated using the GS algorithm, and the resulting light field intensity distribution after passing through the holographic metasurface was simulated (Figure 7d).

## 3 Conclusion

In this work, we introduce a metasurface configuration consisting solely of voxel units made from distinct phases of phase change materials. This metasurface has a single material composition and no complex geometric structure, which greatly simplifies the process complexity during fabrication and modulation compared to traditional metasurfaces. Furthermore, to achieve precise, consistent, and flexible processing and modulation of PCM‐only metasurfaces, we devised a high‐precision voxelated beam deflection 3D fabrication technology leveraging femtosecond laser phase control. Fine deflection control of the femtosecond laser's small spot is achieved by manipulating the blazed grating and Fresnel phases, coupled with a high numerical aperture objective lens. Given our experimental setup, we attain high‐precision PCM‐only metasurface processing, achieving a single‐point modification accuracy below 500 nm and a deflection accuracy under 400 nm. Additionally, we designed a PCM‐only metasurface incorporating a dual‐antenna structure. Utilizing our proposed femtosecond laser phase control method, we achieved precise modulation of the localized writing and erasing of the dual‐antenna units at the “atomic‐subatomic” scale, enabling flexible transitions between “meta‐atoms” and “meta‐molecules” and flexible control over the optical response of metasurface devices. Moreover, to enhance processing efficiency, we shape Gaussian laser into a multi‐spot beam for parallel fabrication, significantly boosting overall efficiency. Ultimately, we showcased a dynamically reconfigurable holographic display and focusing device, both grounded in our PCM‐only metasurface design. The high‐precision pixelated beam deflection technique, facilitated by femtosecond laser phase control, enabled the refresh of holograms and the adjustment of the focal length of the Fresnel zone plate.

Looking forward to the future, the high‐precision pixelated deflection technology based on femtosecond laser phase control proposed in this paper has broad application prospects in the field of micro‐nano fabrication and modulation. In fabrication, the accuracy of feature size is intimately tied to both the beam deflection accuracy and the focused spot size. Consequently, by refining the laser wavelength to a smaller scale using the frequency domain shaping method, we can attain greater deflection accuracy and a reduced diffraction limit, thereby enhancing the feature size accuracy in fabrication. Likewise, utilizing a higher numerical aperture and a shorter focal length objective lens can markedly enhance beam deflection accuracy and diminish the diffraction limit. Furthermore, this technology transcends the limitations of 2D PCM fabrication, leaving ample opportunities for exploration in various materials, including resins and perovskites, as well as in 3D fabrication.

## 4 Experimental Section

### Samples Preparation

Thin‐film samples were prepared by using a radiofrequency magnetron sputtering system (MSP‐300B, Beijing Chuangshi Weina Technology, China) with a high‐purity stoichiometric target. A uniform layer of amorphous Ge_2_Sb_2_Te_5_ (a‐GST) material was transferred from the high‐purity target to the surface of the Al_2_O_3_ substrate at a rate of 0.25 nm s^−1^, resulting in the formation of 50 and 100 nm thick monolayer of a‐GST thin‐film samples. The thickness of the film was verified by using confocal laser light microscopy (OLS5000, Olympus, Japan).

### Laser Processing and Modulation

Spatial light modulators were used to phase control femtosecond lasers for the fabrication of PCM meta atoms. The laser pulses were generated using a commercial fs‐laser system (PHAROS, Light Conversion, Lithuania) which provided a Gaussian distribution of laser pulses with a central wavelength of 1030 nm, a pulse duration of 290 fs, and a repetition rate of 100 kHz. The laser system operated in external trigger mode, with the number of pulses delivered to the sample surface being controlled by an external electrical signal from an independent controller. In the fabrication of crystallization, each pixel was controlled to emit two laser pulses, whereas in the amorphous phase, each pixel was regulated to emit one laser pulse. The fs‐laser pulses were focused on the sample surface using an industrial objective (MPlanFL, 50×/0.80, Olympus, Japan) with 50× magnification and a numerical aperture (NA) of 0.80. A spatial light modulator (PLUTO‐2.1 LCOS Spatial Light Modulator, HOLOEYE) was utilized for femtosecond laser phase modulation. The SLM boasts a resolution of 1920 × 1080, a pixel pitch of 8.0 µm, a fill factor of 93%, and a input frame rate of 60 Hz.

### Characterization and Optical Measurements

The processed images of PCM‐only metasurfaces were studied and characterized using an upright dark‐field optical microscope (BX53, Olympus, Japan) equipped with 50× (MPlanFL N50×/0.8 BD, Olympus, Japan) and 100 × (MPlanFL N100×/0.9 BD, Olympus, Japan) objective lenses and a laser confocal microscope (OLS5000, Olympus, Japan).

### Numerical Simulation

The scattering spectra of the PCM‐only metasurface was modeled using the commercial simulation software FDTD (Lumerical FDTD Solutions) and COMSOL. The complex refractive index of GST was obtained from a previous report.^[^ ^42^ ^]^ The total field/scattered field source was set to impinge on the metasurface at normal incidence from above. Perfectly matched layer boundary conditions were defined around the simulation region.

## Conflict of Interest

The authors declare no conflict of interest.

## Supporting information



Supporting Information

Supplemental Movie 1

Supplemental Movie 2

## Data Availability

The data that support the findings of this study are available in the supplementary material of this article.
