# Supplementary material for: 3D Femtosecond Laser Beam Deflection for High‐Precision Fabrication and Modulation of Individual Voxelated PCM Meta‐Atoms
Source: Adv Sci (Weinh). 2025 Jan 13;12(9):2413316. doi: 10.1002/advs.202413316 (PMC11884528; doi:10.1002/advs.202413316)
Supplement: Supplementary file 1 — Supporting Information [file ADVS-12-2413316-s002.docx]

Supporting Information

Three-Dimensional Femtosecond Laser Beam Deflection for High-Precision Fabrication and Modulation of Individual Voxelated PCM Meta-Atoms

*Weina Han, Donghui Wei, Biye Peng, Jianhui Jiang, Jintao Tong, Zhehao Xu, Xueyan Zou, Jie Hu, Qian Cheng, and Lan Jiang**


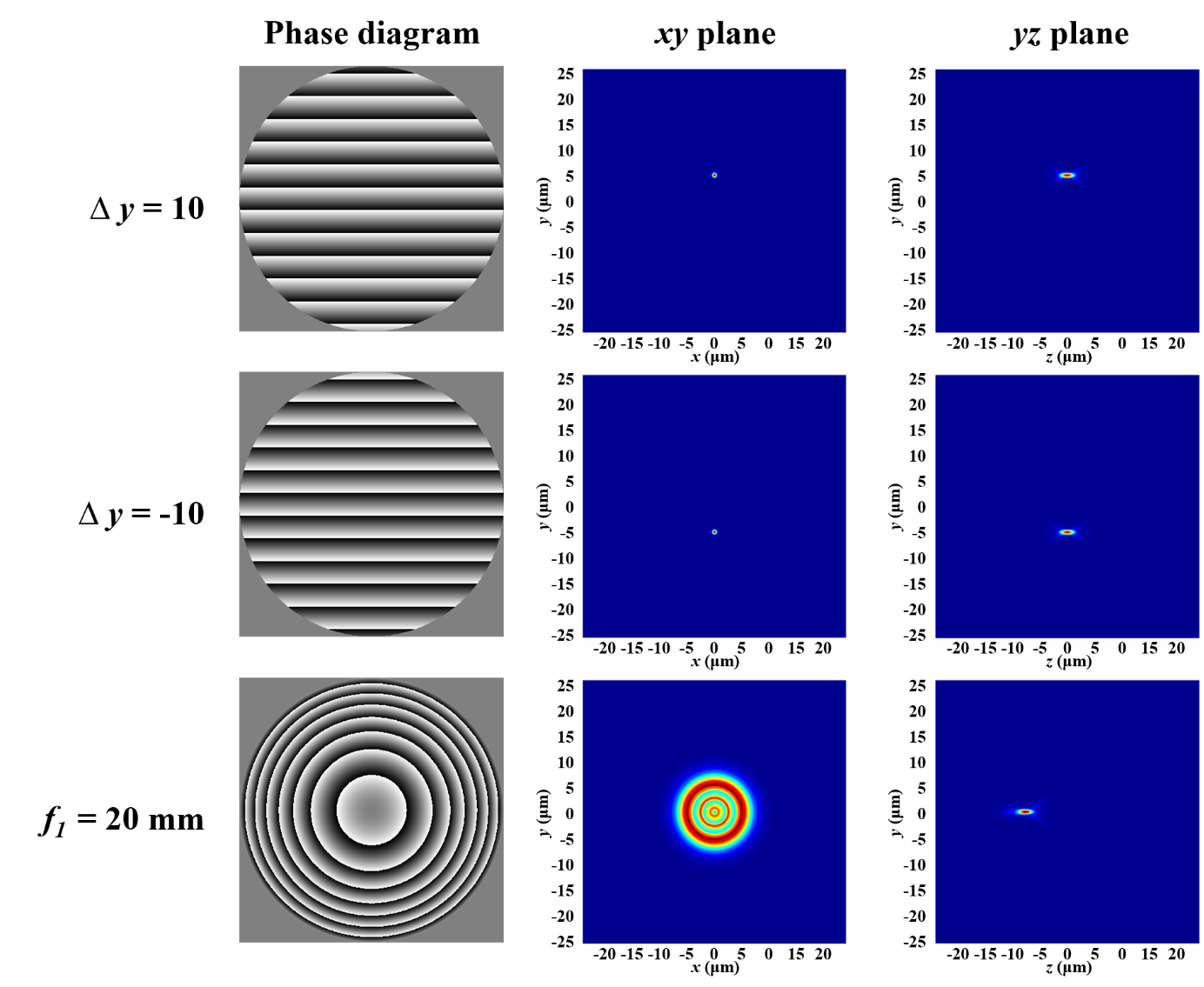


**Figure.S1** Simulation of vector light field propagation of sparkling Grating/Fresnel phase light field.

We simulated the propagation of the phase-controlled deflected beam near the focal plane (objective magnification 50×, numerical aperture NA value 0.8). As shown in Figure S1, we simulated the propagation of the light field through the objective lens for the blazed grating phase of ∆ y = ±10 pixels and the Fresnel lens phase of f = 20mm. It can be found that the method of deflecting and axially displacing the femtosecond laser through phase control can achieve high precision while minimizing the focus spot distortion.


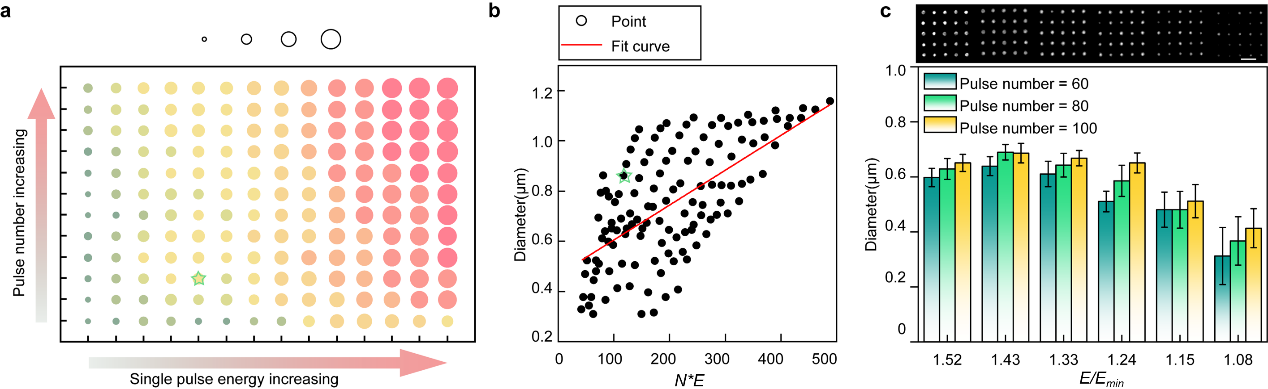


**Figure.S2** Study on fabrication parameters of GST single-point crystallization modification area. (a) Relationship between laser energy, pulse number and modified diameter. (b) Statistics of the relationship between pulse number, pulse energy and modified diameter. (c) Typical pulse number, energy and modified diameter.

To achieve the fabrication of high-precision metasurface units on PCM, a single-point crystallized circle, which constitutes a single voxel of the PCM-based metasurface unit, is essential. The diameter and modification extent of this circle significantly influence the properties of the PCM-only metasurface unit, apart from enabling high-precision deflection. The crystallization of GST film is primarily governed by three factors: the numerical aperture of the objective lens, the energy of each laser pulse, and the number of pulses. Consequently, we investigated the fabrication parameters associated with the single-point crystallization modified circle on a 50nm GST film deposited on a single-crystal silicon substrate, using an objective lens with an NA of 0.8 and a magnification factor of 50×. As shown in Figure S2a, the experimental design entailed varying both the number of pulses and the laser energy from low to high values. The pulse count varied from 20 to 200, with a gradient increment of 20 pulses. Beyond 200 pulses, the gradient was increased to 100 pulses, continuing until 500 pulses were reached. The minimum energy required for crystallization, denoted as *E_min_*, was 1.34nJ, corresponding to a laser pulse intensity that resulted in 20 pulses. The energy gradually escalates until the material undergoes ablation at 3.28nJ. The modified circle exhibits a pronounced contrast variation from the amorphous film when observed under a 100× confocal microscope, attributed to alterations in the refractive index. Consequently, the diameter of the modified circle can be measured for each parameter. For each parameter, 25 data points are recorded, and the average value is calculated after excluding outliers. The statistical outcomes are presented in Figure S2b. A significant positive correlation is observed between the diameter of the modified circle and both the number of pulses and the energy per pulse. The diameter of the modified circle varies within a range of 300 nm to 1200 nm.

To achieve higher fabrication accuracy, the diameter of a single pixel point should be minimized. Nonetheless, excessively focusing on accuracy can compromise processing stability. This is due to the instability of fabricating near the phase change energy threshold, as well as fluctuations in laser energy stability, which collectively contribute to fabricating instability. Consequently, selecting optimal parameters is crucial to achieving both sufficient accuracy and stability. As illustrated in Figure S2c, this study selects three representative pulse numbers: 60, 80, and 100, where the energy is incrementally increased until a stable modified circle is achieved. For clarity, the horizontal axis is denoted as the energy-to-minimum-energy ratio (*E/E_min_*). The stability of the modified circle diameter exhibits a gradual improvement with an increase in both pulse number and energy.


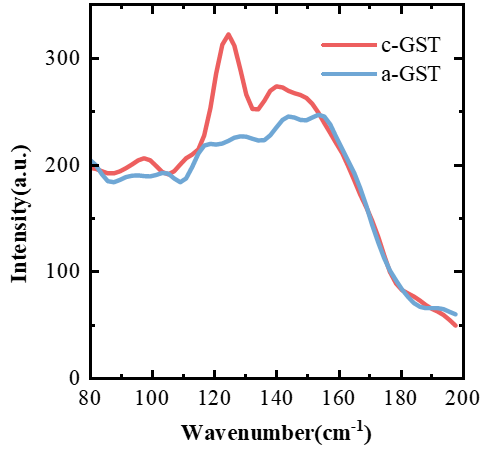


**Figure. S3.** Raman spectra of fabricated and unfabricated areas

By extracting the Raman curves of the processed and unprocessed regions, it can be found that the unprocessed region has a clear amorphous characteristic peak at around 152cm^-1^. The same processed crystalline region exhibits distinct Raman characteristic peaks at around 125 cm^-1^. This indicates that the amorphous GST film has undergone a transition from amorphization to crystallization through femtosecond laser induction.


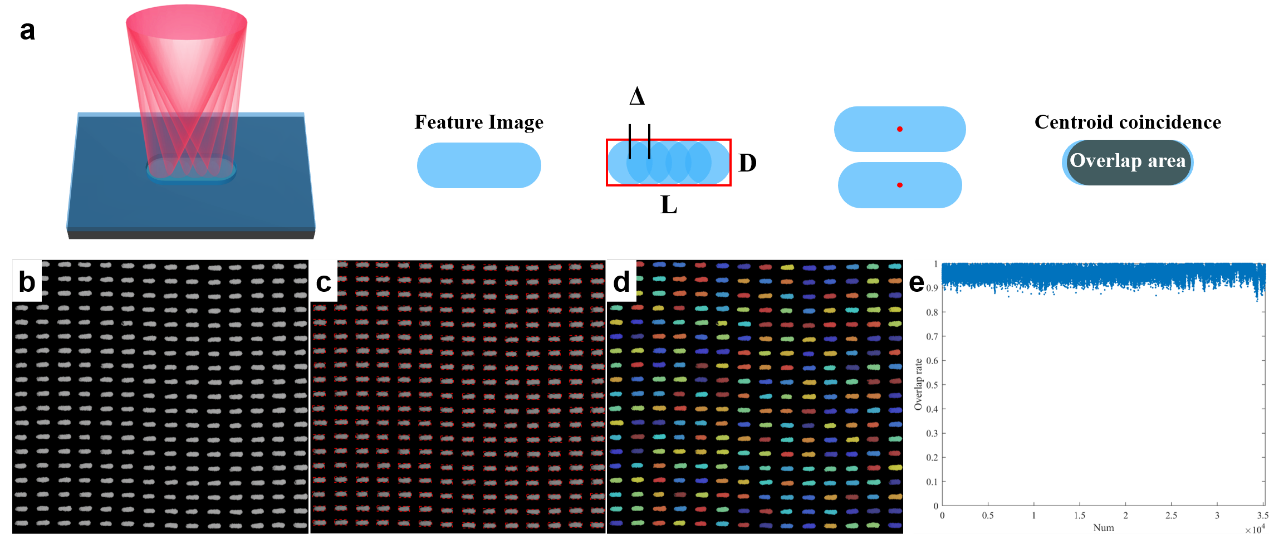


**Figure.S4** (a) Principle of machining accuracy and consistency detection of high-precision voxelization fabrication of metasurfaces based on femtosecond laser phase regulation. (b) Characteristic structure representation. (c) Minimum bounding box extraction of feature structures. (d) Characteristic pattern marking. (e) Consistency.

In order to describe the consistency of the processed structures, this paper will process the overlap between the structures. In order to calculate the overlap rate of the image, this study adopted the following technical routes: image preprocessing, image feature extraction, feature structure marking and overlap rate calculation. The following is a detailed description of each step:

1. Image preprocessing:

Read image: Read raw image data from image file.

Convert color space: If necessary, convert image from RGB color space to a color space more suitable for feature extraction, such as HSV or grayscale.

Adjust brightness and contrast: Adjust the brightness and contrast of image by histogram equalization and other methods to improve the visual effect of image.

2. Image feature extraction:

Edge detection: Use edge detection algorithms (such as Canny, Sobel, etc.) to detect edge information in the image.

Center of mass calculation: For each image, calculate its center of mass coordinates. The center of mass can be obtained by calculating the weighted average position of all pixels in the image, where the weight can be the intensity or label of the pixel.

3. Image alignment:

Center alignment: Align the centroids of two images so that their centroids coincide.

4. Overlap ratio calculation:

Determination of overlap area: On the centroid-aligned images, determine the overlap area of ​​the two images.

Comparison of pixel counts: Calculate the number of pixels in the overlap area and the smaller number of pixels in the two images.

Overlap ratio calculation: Divide the number of pixels in the overlap area by the smaller number of pixels in the two images to obtain the overlap ratio.

Finally, analyze the overlap ratio results and evaluate their consistency with the expected results. Display the overlap ratio results in a chart or numerical form for easy understanding and communication.

As depicted in Figure S4a, a rectangular phase change region is processed via five-pixel deflections, using the high-precision voxel beam deflection technology of the femtosecond laser-controlled super surface. The sample is positioned on the confocal microscope stage, where a 100× objective lens is utilized for observation, and image information is subsequently acquired using the designated 'acquisition' function. The acquired image information is then saved, and the size of the structures is measured utilizing the provided scale data, as demonstrated in Figure S4b. The average size of all structures within the array is calculated, and the deviation of each patterned phase change structure unit from this average is analyzed to determine the mean square error (MSE) of the structure sizes. The characteristic patterned structure is formed by five laser pulses with relative offsets and carefully chosen overlap areas. The characteristic pattern has a length denoted as L, a width as D, and an interval between adjacent deflection pulse lasers represented by Δ. The minimum resolution size, or characteristic size accuracy, of the patterned structure is denoted as D. Image features pertaining to the units of the patterned structure are extracted and appropriately marked. The centroid, pixel area, and other pertinent data of the patterned structure features are quantified. A 19×14 array of the patterned phase change structures is identified, and the corresponding minimum bounding box is provided, as illustrated in Figure S4c.

The pixel values for the length and height of each minimum bounding box are tallied, and the dimensional accuracy of all patterned structures is subsequently determined based on the relevant scale. The average length of the patterned structures is 1.7739 μm, while the average height is 0.7306 μm. Consequently, the statistical feature size accuracy amounts to 0.7306 μm. The mean square error (MSE) of the length data for each patterned phase change unit is 10.9 nm, indicating a size deviation of 10.9 nm. By aligning the centroids of any two imaged features, the overlapping pixel area between the two patterned features is quantified, allowing the overlap rate to be expressed as the ratio of the overlapping pixel area to the minimum area of the two features. By assessing N patterned structures, N*(N-1)/2 overlap rate data points are acquired, and the average overlap rate is subsequently derived through calculation, providing the consistency index for the patterned structure unit. The features of the patterned structural units were extracted, and a 19×14 array of patterned phase change structures was marked (as shown in Figure S4d). Additionally, the centroids and pixel areas of all units were calculated (as shown in Figure S4c). By aligning the centroids of any two image features and quantifying the overlapping pixel areas between the two patterned features, 35,245 sets of overlapping rate data were collected (as shown in Figure S4e), yielding a statistical average of 96%.


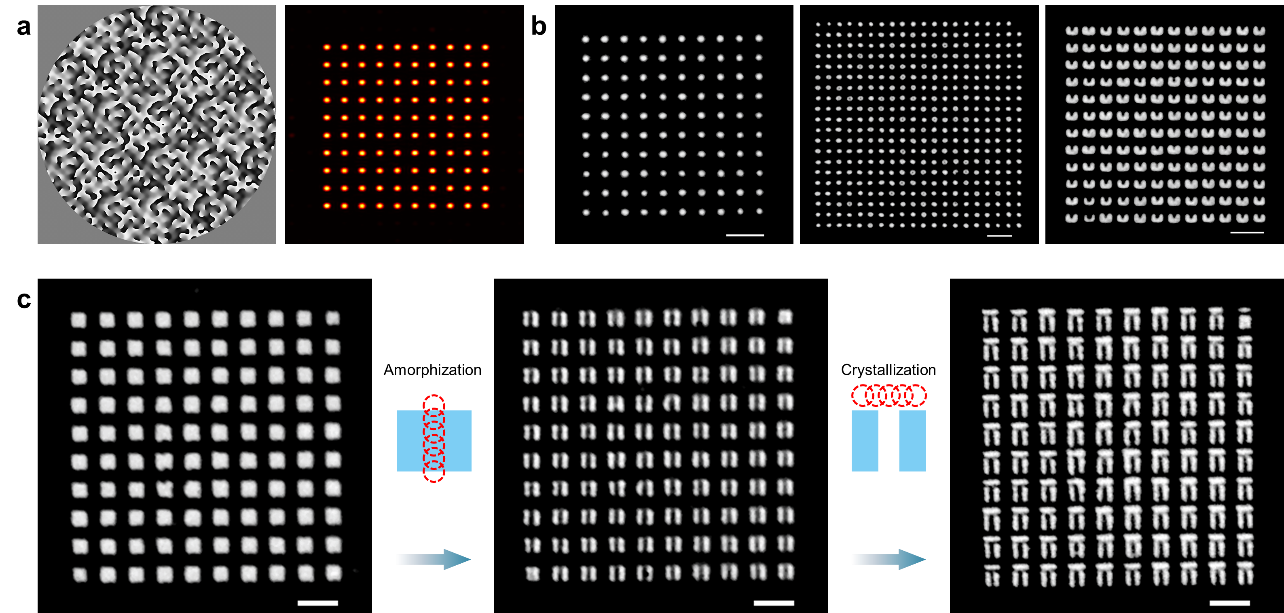


**Figure.S5** Femtosecond laser multi-spot parallel fabrication and modulation. (a) Multi-spot phase diagram and multi-spot vector light field simulation diagram. (b) Optical micrographs of multi-spot single-point processing and patterning processing. (c) Dynamic refreshing of the metasurface is achieved based on “sub-meta-atom” level modulation of multiple-spot.

In contrast to point-by-point fabrication, parallel fabrication fully harnesses single laser energy, transitioning from a single light spot to multiple light spots, leading to a marked enhancement in processing efficiency. The application of the weighted GS algorithm enables precise calculation of the phase hologram for a two-dimensional light field featuring arbitrary intensity distributions. Figure S5a illustrates the hologram at the SLM plane, calculated using the GS algorithm with a wavelength of 1030nm, 1080×1080 SLM pixels, and a pixel period of 8μm. Additionally, it depicts the light field intensity distribution at the focal plane of a 50× objective lens with an NA value of 0.8. Within this configuration, the multi-spot array is arranged in a 10×10 grid with a spacing of 6-pixel intervals. Figure S5b displays the 10×10 and 20×20 multi-spot crystallization lattices, along with a 12×12 multi-spot deflection pattern fabrication on the GST sample, arranged from left to right. Figure S5c demonstrates the use of a 10×10 multi-light spot array to parallelly fabricate the square atomic configuration of the PCM-only metasurface. Through pixel-level deflection, subatomic-level amorphization erase modulation and crystallization write modulation were applied to the atomic configuration. Ultimately, we achieved a 10×10 meta-molecular configuration metasurface array featuring a three-atom configuration, marking a remarkable two-order-of-magnitude improvement in fabrication efficiency.


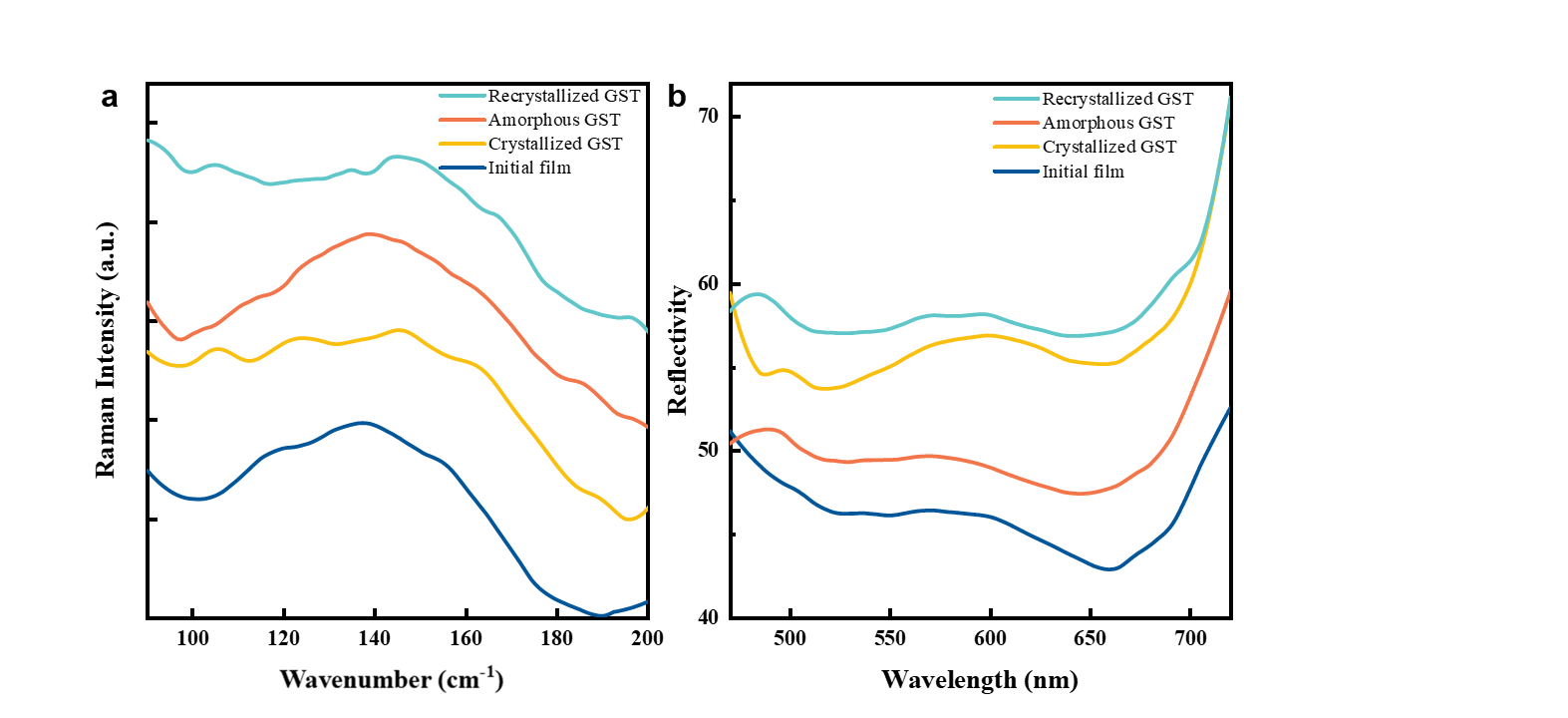


**Figure.S6** The changes in optical properties of GST during the crystallization amorphization recrystallization process. (a) Raman spectroscopy. (b) Visible light band reflectance spectrum.

As shown in Figure S6, we characterized the spectral response of GST during the crystallization amorphization recrystallization modulation process on a sapphire substrate GST sample with a film thickness of 100 nm. Figure S6a shows the Raman spectrum curve of GST reversible modulation. The initial Raman spectral characteristics of aGST thin film are mainly characterized by a large envelope peak at 140cm^-1^, which is related to the SbTe bond vibration in the Sb_m_Te_3_ (*m*=1,2) tetrahedral lattice unit, confirming the amorphous phase of GST. After the first crystallization of GST,^[1-2]^ significant peaks appeared at 105cm^-1^ and 125cm^-1^, corresponding to the softened Al mode of the angle shared GeTe_4_ tetrahedron and the GeTe_6_ octahedral vibration mode, respectively, proving the transition of GST phase from amorphous to metastable crystal (face centered crystal, fcc) state.^[3-4]^ After a single pulse of femtosecond laser was applied in the crystallization region, the peaks at 105cm^-1^ and 125cm^-1^ significantly disappeared, and transformed into a large envelope broad peak at 140cm^-1^, proving its amorphous phase. When the femtosecond laser was written again, the peak at 105cm^-1^ significantly increased again, indicating that GST had transformed from amorphous phase to crystalline phase again.

Figure S6b shows the reflectance variation in the visible light band during the GST dynamic reversible modulation process. It can be observed that the reflectance undergoes cyclic changes from increasing to decreasing and then to increasing during the crystallization amorphization recrystallization process, demonstrating that our device can achieve dynamic control of optical properties.

**Movie S1.** Multi-spot parallel efficient fabrication.

This video shows the experiment presented in Figure S5. Movie S1 displays the 10×10 multi-spot crystallization and along with a 12×12 multi-spot deflection pattern fabrication on the GST sample. (The video is processed at four times the speed)

**Movie S2.** Reconfigurable Fresnel zone plate.

This video shows the dynamic reconfiguration of the focal length of the Fresnel zone plate device. Modulation of Fresnel zone plate focal length from 20 μm to 50 μm through the process of writing-erasing-rewriting on GST film. (The modulation process in the video is played at 200× speed.)

References

[1]J. Du, Z. Mu, L. Li, J. Li, *Optics & Laser Technology* **2021**, *144*, 107393.

[2]H. Yoon, W. Jo, E. Cho, S. Yoon, M. Kim, *Journal of non-crystalline solids* **2006**, *352* (36-37), 3757.

[3]M. V. Rybin, A. D. Sinelnik, M. Tajik, V. A. Milichko, E. V. Ubyivovk, S. A. Yakovlev, A. B. Pevtsov, D. A. Yavsin, D. A. Zuev, S. V. Makarov, *Laser & Photonics Reviews* **2022**, *16* (2), 2100253.

[4]W. Han, Y. Dai, D. Wei, X. Zhang, L. Han, B. Peng, S. Jiao, S. Weng, P. Zuo, L. Jiang, *ACS Appl. Mater. Interfaces* **2024**, *16* (2), 2836.
